# Structural basis of p53 inactivation by cavity-creating cancer mutations and its implications for the development of mutant p53 reactivators

**DOI:** 10.1038/s41419-024-06739-x

**Published:** 2024-06-11

**Authors:** Dimitrios-Ilias Balourdas, Anja M. Markl, Andreas Krämer, Giovanni Settanni, Andreas C. Joerger

**Affiliations:** 1 Institute of Pharmaceutical Chemistry, Goethe University, Max-von-Laue-Str. 9, 60438 Frankfurt am Main, Germany; 2 Structural Genomics Consortium (SGC), Buchmann Institute for Molecular Life Sciences, Max-von-Laue-Str. 15, 60438 Frankfurt am Main, Germany; 3 Faculty of Physics and Astronomy, Ruhr University Bochum, Universitätsstr. 150, 44801 Bochum, Germany; 4 Physics Department, University of Mainz, Staudingerweg 7, 55099 Mainz, Germany

**Keywords:** Tumour-suppressor proteins, X-ray crystallography, Target identification

## Abstract

The cavity-creating p53 cancer mutation Y220C is an ideal paradigm for developing small-molecule drugs based on protein stabilization. Here, we have systematically analyzed the structural and stability effects of all oncogenic Tyr-to-Cys mutations (Y126C, Y163C, Y205C, Y220C, Y234C, and Y236C) in the p53 DNA-binding domain (DBD). They were all highly destabilizing, drastically lowering the melting temperature of the protein by 8–17 °C. In contrast, two non-cancerous mutations, Y103C and Y107C, had only a moderate effect on protein stability. Differential stabilization of the mutants upon treatment with the anticancer agent arsenic trioxide and stibogluconate revealed an interesting proximity effect. Crystallographic studies complemented by MD simulations showed that two of the mutations, Y234C and Y236C, create internal cavities of different size and shape, whereas the others induce unique surface lesions. The mutation-induced pockets in the Y126C and Y205C mutant were, however, relatively small compared with that of the already druggable Y220C mutant. Intriguingly, our structural studies suggest a pronounced plasticity of the mutation-induced pocket in the frequently occurring Y163C mutant, which may be exploited for the development of small-molecule stabilizers. We point out general principles for reactivating thermolabile cancer mutants and highlight special cases where mutant-specific drugs are needed for the pharmacological rescue of p53 function in tumors.

## Introduction

The tumor suppressor p53 is inactivated by mutation in about half of all cancer cases. Reactivation of mutant p53 is, therefore, a prime target in the development of novel cancer therapies [1–3]. Most p53 cancer mutations are missense mutations clustering in the DNA-binding domain (DBD). Even though there are several hotspot mutations in the DBD, the p53 cancer mutome as a whole is complex and diverse, with more than 2000 different, largely uncharacterized p53 missense mutants observed in tumors [4, 5]. The missense mutations can roughly be classified as either contact mutations that remove essential DNA-contact residues or structural mutations that lower the thermodynamic and kinetic stability of the DBD [6]. Due to its low intrinsic stability, the human p53 DBD is very susceptible to inactivation by destabilizing mutations, resulting in unfolding, followed by rapid aggregation at body temperature [7]. Many structural mutants are temperature-sensitive (ts): they are inactive at body temperature but transcriptionally active at subphysiological temperatures [8–11]. This observation has important implications for potential mutant reactivation strategies via so-called chemical chaperones. In principle, any molecule that binds to the native but not the denatured state of the mutant should shift the folding-unfolding equilibrium toward the folded state and potentially restore a wild-type-like activity. Such chemical chaperones could either act as generic stabilizers, potentially rescuing multiple mutants, or target a mutant-specific lesion on the surface. In a landmark study, Chen and colleagues showed that many—but not all—structural mutants can be reactivated with arsenic trioxide (ATO), which allosterically stabilizes the DBD by covalently coordinating three cysteines in a cryptic binding site in the loop-sheet-helix motif [12]. In a related follow-up study, it was shown that antimony noncovalently binds to the same three cysteines but reactivates fewer ts mutants than arsenic trioxide overall [13]. More recently, a small molecule was discovered by computation-based methods that is suggested to bind to a cryptic pocket in the same region, and reactivation was reported for several mutants, including, somewhat surprisingly, also DNA-contact mutants [14]. A binding mode has been proposed from docking studies but has not yet been experimentally confirmed. Other strategies aim at reactivating zinc-binding deficient mutants with synthetic metallochaperones via an increase in cellular zinc levels [15, 16].

We have previously shown that the Y220C mutant is an ideal test case for the development of mutant-specific small-molecule stabilizers. This highly destabilizing mutation creates an extended, druggable crevice on the surface [17]. We have mapped key interaction sites in this pocket for designing potent binders [18–21] and developed a series of small molecules that specifically bind to this crevice, stabilize the protein, inhibit aggregation, and, most importantly, restore p53 signaling in Y220C mutant bearing cancer cell lines [22, 23]. Building on this work, PMV Pharmaceuticals have developed a Y220C-reactivating molecule, PC14586 (rezatapopt), which is currently in clinical trials [24].

Our work on the Y220C and the related Y220S and Y220N cancer mutants [25] prompted us to search the p53 cancer mutome for other potentially druggable mutants with a mutation-induced pocket. Here, we have systematically analyzed the energetic and structural effects of other cancer-related Tyr-to-Cys mutations in the p53 DBD as such mutations are (i) relatively frequent and (ii) poised to induce the formation of a cavity. And indeed, all cancer-associated variants were highly destabilizing, albeit to a varying degree, and induced unique cavities, with the size and accessibility of the pocket varying, depending on the structural context of the mutation. The differential effect on the stability of the Tyr-to-Cys mutants observed upon treatment with ATO highlighted the need for mutant-specific rescue drugs for some variants, making a strong case that the development of generic and mutant-specific stabilizers should go hand in hand to successfully target the druggable p53 mutome and develop potent mutant reactivators for cancer therapy.

## Results and discussion

### Frequency and location of Tyr-to-Cys cancer mutations in the p53 DBD

**Fig. 1:**
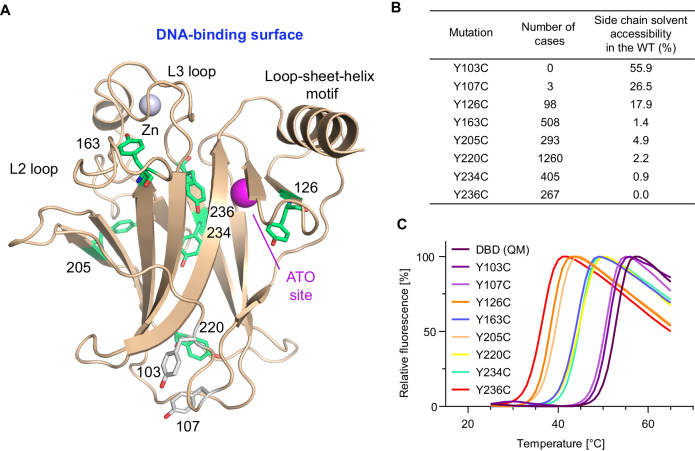
Location of Tyr-to-Cys cancer mutations in the p53 DBD. **A** Cartoon representation of the human p53 DBD (PDB entry 2XWR; [66]) with all eight tyrosine residues in the DBD highlighted as stick models. Six of those tyrosines (colored in green) are frequently mutated into a cysteine in cancer. The magenta sphere marks the location of the cryptic arsenic trioxide binding site. **B** Number of cases in the 2017 release of the UMD TP53 Database (*N* = 80,402) and solvent accessibility of each tyrosine in the structure of the wild-type DBD, calculated using NACCESS with chain A of PDB entry 2XWR [66]. **C** Differential effect of Tyr-to-Cys mutations on the thermal stability of the p53 DBD. Shown are normalized DSF melting curves of a stabilized p53 DBD (QM, M133L/V203A/N239Y/N268D) and its eight Tyr-to-Cys mutant variants recorded at a heating rate of 3 °C/min. See also Table 1 for a list of *T*_m_ values obtained upon analysis.

Tyr-to-Cys mutations are potentially cavity-creating. There are eight tyrosine residues in the human p53 DBD, spread across the whole domain (Fig. 1A). We systematically analyzed the frequencies of cancer mutations at these codons in the UMD TP53 Database [26] (https://p53.fr/tp53-database) and the IARC/NCI TP53 Database [4] (https://tp53.isb-cgc.org) (Fig. 1B and Supplementary Tables S1, S2). Y220C is the most frequent mutation, ranking at no. 7 or 9 in the two databases, and may currently account for up to 150,000 new cancer cases per year worldwide. This number is estimated based on (i) the relative frequency of this mutation in the UMD database, (ii) the assumption that p53 is mutated in every second tumor, and (iii) a worldwide cancer incidence of 19.3 million in 2020 [27]. Y163C, Y205C, Y234C, and Y236C are also frequently found in cancer, all ranking within the top 40 cancer-associated p53 missense mutations and collectively accounting for close to 180,000 new cancer cases per year. The Y234C mutation is particularly prevalent in chronic lymphocytic leukemia, where it has been associated with chlorambucil treatment [28]. Y126C is less frequent but still occurs with an appreciable frequency. Tyr103 and Tyr107, however, are mutated only very rarely in human cancer, if at all. Incidentally, these two tyrosine residues display the highest solvent-accessibility in the wild-type structure (Fig. 1B). In addition to mutation to a cysteine, there are also cancer-associated variants with a histidine, serine, asparagine, or aspartate in each case, but they occur with a much lower frequency. Of note, the Y107H mutation is a fairly frequent germline variant in the African population [29] and has recently been described as hypomorphic [30]. There are also a few cases of conservative Tyr-to-Phe mutations in both databases, which are, in most cases, likely to be passenger mutations. A notable exception seems to be the Y205F variant, which was found to be inactive in the recent functional annotation of the p53 mutome [31] and is incidentally also more frequent than the other Tyr-to-Phe mutants.

### Differential effect of Tyr-to-Cys mutations on protein stability

To determine the effect of the mutations on the thermostability of the p53 DBD, the Tyr-to-Cys mutations were introduced into a stabilized quadruple mutant variant (M133L/V203A/N239Y/N268D), which we have routinely used for biophysical and structural studies of conformationally unstable p53 mutants in the past [17, 25, 32]. In the case of the Y205C mutant, we also used a triple mutant variant without the V203A substitution (M133L/N239Y/N268D) because the side chain of residue 203 lines the anticipated pocket created by the Y205C mutation. The melting temperature (*T*_m_) of the mutant DBDs was determined by differential scanning calorimetry (DSF), showing that all cancer-associated Tyr-to-Cys mutants were highly destabilized, with the *T*_m_ of the DBD lowered by 8 to 17 °C (Table 1 and Fig. 1C), suggesting that they are all largely unfolded in cells at body temperature, as shown previously for the Y220C mutant [33]. The drastically different stability loss induced by the Y234C and Y236C mutation (Δ*T*_m_ of 8 vs 17 °C) is particularly interesting, given that the two mutations affect the same region of the hydrophobic core. The Y103C and Y107C mutations had only a minor destabilizing effect, reducing the *T*_m_ by only about 2 °C, which explains why they are not selected in cancer.

**Table 1:** Melting temperatures of p53 mutant DBDs.

Tyr mutation	*T*_m_ (°C)^a^	Δ*T*_m_ (°C)^b^
WT (QM)	51.9 ± 0.3 (5)	-
WT (TM)	50.6 ± 0.1 (4)	-
Y103C	50.5 ± 0.3 (5)	−1.4
Y107C	49.9 ± 0.3 (5)	−2.0
Y126C	36.9 ± 0.2 (3)	−15.0
Y163C	43.0 ± 0.2 (3)	−8.9
Y205C	37.8 ± 0.2 (3)	−14.1
Y205C (TM)	36.5 ± 0.2 (3)	−14.1
Y220C	43.3 ± 0.3 (5)	−8.6
Y234C	43.9 ± 0.3 (5)	−8.0
Y236C	35.1 ± 0.3 (3)	−16.8

^a^Melting temperatures of Tyr-to-Cys mutants were measured in the framework of a stabilized quadruple mutant DBD (QM; M133L/V203A/N239Y/N268D), and for the Y205C mutant also in the framework of a stabilized triple mutant (TM; M133L/N239Y/N268D). The *T*_m_ of the wild-type DBD is ~45 °C [63, 64]. Buffer: 25 mM HEPES, pH 7.5, 500 mM NaCl, 0.5 M TCEP. The number of independent experiments is shown in parentheses, mean ± SD is given.

^b^Δ*T*_m_ = *T*_m_ (Tyr-to-Cys mutant) - *T*_m_ (corresponding wild-type construct).

### Structural effects of mutation: Y234C and Y236C create internal cavities of different size

We determined crystal structures of all six cancer-associated Tyr-to-Cys DBD mutants at a resolution ranging from 1.38–2.10 Å (Table 2). In all cases, the overall structural features of the wild-type DBD were conserved: a central, antiparallel ß-sandwich and an extended DNA-binding surface formed by a loop-sheet-helix motif and two large loops (L2 and L3) that are stabilized via zinc coordination.

**Table 2:** X-ray data collection and refinement statistics.

Mutant	Y126C (QM)	Y163C (QM)	Y205C (TM)	Y220C (QM)	Y234C (QM)	Y236C (QM)
*Data Collection*						
Space Group	*P*6_5_22	*C*222_1_	*P*2_1_2_1_2_1_	*P*6_5_22	*P*2_1_2_1_2_1_	*P*2_1_2_1_2_1_
*a* (Å)	45.19	68.60	64.96	45.33	65.06	65.15
*b* (Å)	45.19	84.50	70.90	45.33	70.99	71.06
*c* (Å)	331.30	65.68	105.06	332.64	105.13	105.26
*α* (°)	90	90	90	90	90	90
*β* (°)	90	90	90	90	90	90
*γ* (°)	120	90	90	120	90	90
Molecules/AU	1	1	2	1	2	2
Resolution (Å)^a^	39.13–1.69 (1.72–1.69)	42.25–1.65 (1.68–1.65)	47.90–1.54 (1.54–1.57)	47.52–1.44 (1.46–1.44)	47.96–1.38 (1.40–1.38)	43.69–2.10 (2.16–2.10)
Unique reflections	24,105	23,257	72,298	38,726	100,451	28,446
Completeness (%)^a^	99.9 (99.9)	99.6 (98.7)	99.8 (99.8)	99.4 (94.1)	99.9 (99.9)	97.8 (99.3)
Multiplicity^a^	7.2 (7.7)	5.5 (4.0)	6.6 (6.5)	13.2 (13.2)	6.6 (6.8)	5.4 (5.5)
*R*_meas_ (%)^a^	5.3 (93.2)	6.9 (66.7)	5.1 (82.0)	4.7 (113.7)	5.3 (93.8)	20.0 (105.2)
CC(1/2)^a^	0.999 (0.803)	0.999 (0.815)	0.999 (0.893)	1.000 (0.907)	0.999 (0.874)	0.994 (0.764)
Mean *I/σ(I)*^a^	17.6 (2.4)	13.1 (2.0)	15.9 (2.0)	28.0 (2.5)	17.1 (2.3)	6.7 (2.0)
*Refinement*						
*R*_work_, (%)^b^	19.3	16.7	18.4	16.6	15.2	21.9
*R*_free_, (%)^b^	22.2	19.2	22.0	21.1	17.7	26.4
No. of atoms						
Protein^c^	1482	1543	3094	1476	3135	3090
Zinc	1	1	2	1	2	2
Water	145	199	361	184	562	334
Other ligands	4	15	52	1	12	24
RMSD bonds (Å)	0.006	0.006	0.005	0.005	0.005	0.007
RMSD angles (°)	0.79	0.84	0.80	0.75	0.77	0.87
Mean *B* (Å^2^)	31.2	23.9	28.1	29.3	21.2	25.4
PDB entry	8QWK	8QWL	8QWM	8QWN	8QWO	8QWP

^a^Values in parentheses are for the highest-resolution shell.

^b^*R*_work_ and *R*_free_ = ∑||*F*_obs_|- |*F*_calc_ | |/∑|*F*_obs_|, where *R*_free_ was calculated with 5% of the reflections chosen at random and not used in the refinement.

^c^Number includes alternative conformations.

**Fig. 2:**
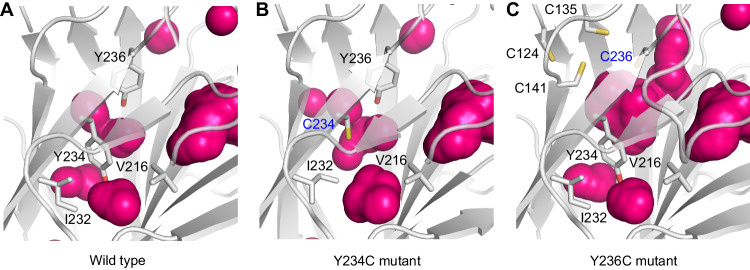
Internal cavities in the p53 DBD induced by the oncogenic Y234C and Y236C mutations. Cavities in the hydrophobic core of the DBD are shown as red spheres calculated with PyMOL using a cavity detection radius and cavity detection cutoff set at 5 solvent radii. **A** Wild type (PDB entry 1UOL), **B** Y234C mutant, and **C** Y236C mutant. Mutation sites are highlighted in blue. The Y234C mutation induces a modest expansion of small preexisting cavities, with a flip of the Ile232 side chain filling some of the void created by the loss of the aromatic side chain of Tyr234. In contrast, the Y236C mutation creates a much larger internal cavity without the collapse of neighboring side chains into the pocket. See also Table 3 with the solvent-accessible volume of the largest internal pocket at each mutation site.

The crystal structures of the Y234C and Y236C mutants revealed that both mutations induce a substantial loss of hydrophobic interactions, resulting in the formation/expansion of internal cavities in the hydrophobic core of the DBD, without affecting its overall architecture (Fig. 2), and, as such, these mutants represent classical ts mutants. In the Y234C mutant, the side chain of Ile232, next to Cys234, was flipped relative to its orientation in the wild type, with the CD atom extending into the void created by the large-to-small mutation. Minor shifts of 0.7–0.9 Å were also observed for the side chain of Val197 lining the pocket. The net result is a modest expansion of the internal cavity volume near the space previously occupied by the phenolic side chain of Tyr234. The cavity created by the Y236C mutation was significantly larger compared with that induced by the Y234C mutation (Fig. 2 and Table 3), which provides a rationale for the much more severe loss in thermal stability upon the Y236C mutation. Interestingly, the sulfur atom of the mutated residue, Cys236, is located almost within the van der Waals distance of the sulfur atoms of Cys135 and Cys141 (distance of 4.1 and 4.7 Å, respectively). The latter two cysteines, together with the adjacent Cys124, have been associated with conformational instability/plasticity of the human p53 DBD [34] and form a cryptic binding site for arsenic [12] and antimony [13].

**Table 3:** Size of mutation-induced cavities in the p53 DBD.

Mutant Structure	Solvent-accessible Area (Å^2^)^a^	Solvent-accessible Volume (Å^3^)^a^
*Surface crevices*		
Y220C, entry 6SHZ, chain A	73.5	14.7
Y220C, entry 6SHZ, chain B	98.9	24.3
Y220C, hexagonal crystal form	103.4	31.7
Y126C, conformation A^b^	16.7	2.3
Y126C, conformation B^b^	26.0	5.9
Y205C, chain A	19.5	3.3
Y205C, chain B	24.3	4.8
Y163C, crystal	35.6	8.8
Y163C, MD snapshot f35486	73.6	39.7
*Internal cavities*		
Y234C, chain A^c^	11.3	1.6
Y234C, chain B	11.2	1.6
Y236C, chain A^c^	30.6	7.3
Y236C, chain B	32.4	8.1

^a^Solvent-accessible area and solvent-accessible volume of the mutation-induced pockets were calculated using the CASTp webserver [65] (probe radius of 1.4 Å). For the crystal forms with two molecules in the asymmetric unit, the values for both chains are given.

^b^Cys126 adopts two alternative conformations in the crystal structure, with conformer A having a higher occupancy than conformer B.

^c^Parameters for the largest internal cavity at the mutation site are given. In the structure of the corresponding pseudo-wild-type DBD (PDB entry 1UOL), only much smaller cavities were detected next to the Tyr234 or Tyr236 side chain, with a maximal solvent-accessible volume of 0.34 Å^3^ for the pocket between the Tyr234 hydroxyl and Val216 (see Fig. 2).

**Fig. 3:**
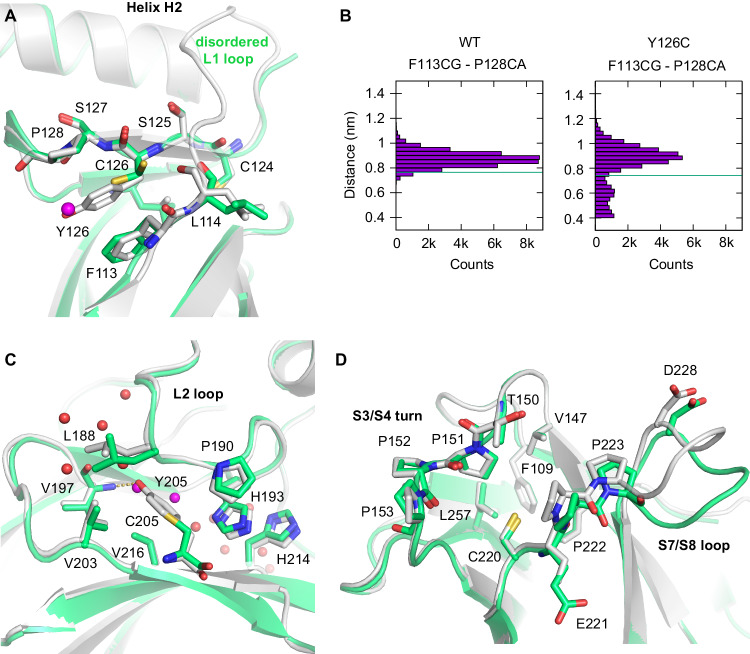
Mutation-induced surface crevice in the structures of the Y126C and Y205C cancer mutant. **A** Loop-sheet-helix motif region of the Y126C mutant (green) superimposed onto that of the corresponding wild-type DBD (gray; PDB entry 1UOL). Cys126 in the mutant structure adopts two alternative conformations. A mutant-specific structural water molecule in the created cavity next to Cys126 is shown as a magenta sphere. **B** Distance between the CG atom of Phe113 and the CA atom of Pro128 in MD simulations, showing a multimodal distribution in the Y126C mutant. The green line indicates the distance in the corresponding crystal structure (“WT” = chain A of PDB entry 1UOL). **C** View of the mutation site in the structure of the Y205C DBD (green model) superimposed onto the wild-type DBD (gray; PDB entry 2XWR). Selected side chains around the mutation site are shown as stick models. The hydrogen bond between the phenolic hydroxyl of Tyr205 in the wild type and the backbone amide of Val197 is highlighted with an orange broken line. Selected structural water molecules in the mutant structure are shown as red and magenta spheres. Two water molecules colored in magenta are unique to the mutant structure and occupy positions that are only accessible due to the mutation to a smaller side chain. **D** Mutation-induced pocket in the Y220C mutant in different crystal forms. A superimposition of the structures of the Y220C mutant in the hexagonal crystal form (green) and the orthorhombic crystal form (gray; PDB entry 6SHZ, chain A) shows that a more open state of the pocket is stabilized in the hexagonal crystal packing.

The other Tyr-to-Cys mutations created characteristic surface lesions, resulting in surface crevices of varying size and depth (Table 3). The structure of the Y220C mutant in a new, hexagonal crystal form showed a slightly more open state of the mutation-induced surface crevice, due to shifts of the S7/S8 loop around Pro222 and Pro223, compared with the previously determined structure in a different crystal form (Fig. 3D). These differences reflect the intrinsic plasticity of this pocket, consistent with earlier molecular dynamics (MD) simulations and structures of ligand-bound Y220C mutant [20].

### Y126C and Y205C mutations induce small water-filled pockets on the surface

The Y126C mutation induced a small cavity on the surface of the loop-sheet-helix motif (Fig. 3A). In the wild-type structure, the side chain of Tyr126 is sandwiched between Pro128 and Phe113, forming stabilizing hydrophobic and π-interactions. These interactions are lost in the mutant, explaining the high stability loss. The side chain of Cys126 adopts two alternative conformations in the crystal structure, one of which is pointing in the direction of the phenolic group in the wild type, whereas the other, occurring with lower occupancy, faces the backbone of Leu114. Residues 115–120 in the neighboring L1 loop could not be unambiguously modeled, indicating a high conformational flexibility of this region. However, it has to be noted that the structure of the Y126C mutant was solved in a crystal form in space group P6_5_22 that is generally associated with a disordered L1 loop, also in the case of distant mutation sites (e.g., for the R249S mutant in PDB entries 2BIO and 3D08 [35, 36], and the Y220C mutant structure in this study). MD simulations of the Y126C mutant showed an increased structural plasticity around the mutation site, with alternative conformational states occurring where Phe113 collapsed into the pocket but also more open states characterized by a flip of the Phe113 side chain towards Cys124 (Fig. 3B and Supplementary Figs. S1, S3A). The flip of Phe113, however, was also observed for the wild-type reference (Supplementary Fig. S1B).

Tyr205 is located on β-strand S6 on the edge of the β-sandwich, with its aromatic side chain packing against the L2 loop. It forms hydrophobic interactions with Leu188, Pro190, Val203, and Val216, as well as a hydrogen bond with the backbone amide of Val197 (Fig. 3C). Most of these interactions are lost in the crystal structure of the Y205C mutant, accounting for the high stability loss, and there is virtually no collapse of the created cavity, apart from minor rearrangement of the Leu188 side chain. Two additional structural water molecules occupy the space created by the large-to-small substitution, which effectively links two narrow water channels that preexist in the wild-type structure on either side of the phenolic group of Tyr205 (Fig. 3C).

### Dynamic mutation-induced pocket in the Y163C mutant DBD

**Fig. 4:**
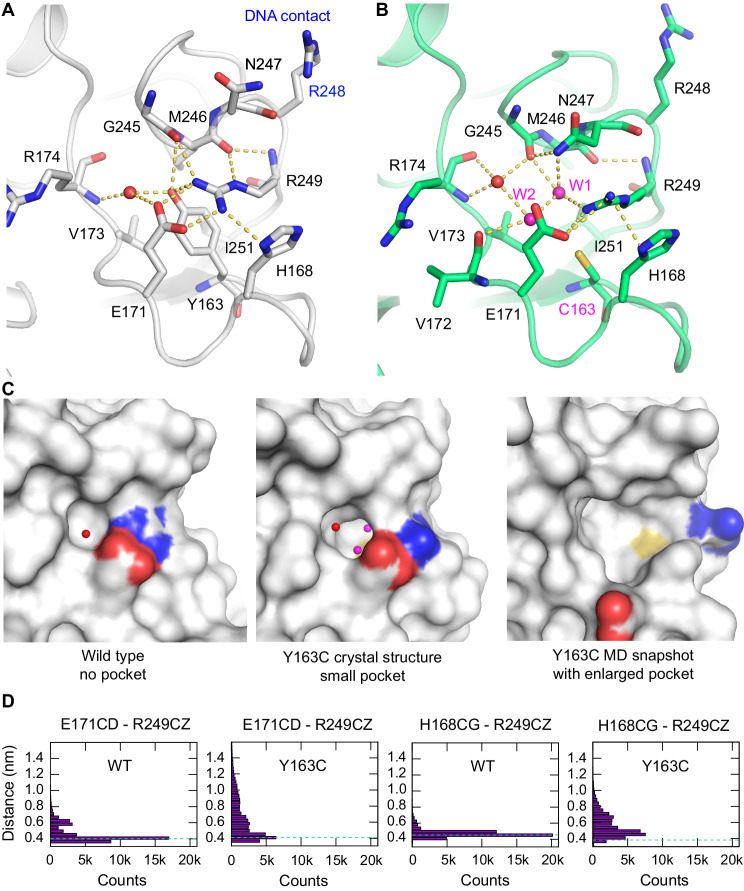
Local conformational changes and formation of a pocket in the p53 DBD upon Y163C mutation. **A** Interaction network around Tyr163 in the wild type. Key residues are shown as stick models, and selected hydrogen bonds are highlighted with yellow broken lines. A structural water molecule interacting with the phenolic side chain of Try163 is shown as a red sphere. **B** Structural rearrangements in the Y163C mutant are shown in the same orientation as the wild type in panel **A**. The mutation-induced cavity creates space for two additional structural water molecules, shown as magenta spheres. **C** Molecular surface around residue 163 in the wild type and the Y163C mutant, revealing the extent and depth of the mutation-induced pocket. The carboxylate oxygens of Glu171 are shown in red, the guanidinium group nitrogen atoms of Arg249 in blue, and the sulfur atom of Cys163 in yellow. A representative snapshot from the MD simulation (surface on the right; snapshot f35486, at time point 106.88 ns of MD run no. 4) shows the breaking of the Glu171-Arg249 salt bridge, which results in a drastic enlargement of the mutation-induced cavity (see also Table 3 and Supplementary Figs. S2, S3). The orientation is similar to the one in panels (**A**, **B**). **D** Distribution of selected distances involving Arg249 in MD simulations. The green dashed line indicates the distance in the corresponding crystal structure (“WT” = chain A of PDB entry 1UOL).

The structure and dynamics of the Y163C mutant DBD are particularly interesting. The side chain of Tyr163 plays an important role in stabilizing the conformation of the L2/L3 loop region, which docks to the minor groove of DNA response elements via Arg248. The guanidium group of Arg249 stabilizes a hairpin-like, binding-competent conformation of the L3 loop via a salt bridge with Glu171 and hydrogen bonds with the backbone oxygens of Gly245 and Met246 (Fig. 4A). This orientation is further stabilized via a cation-π interaction of the guanidinium group with the side chain of Tyr163, which in turn packs against the hydrophobic side chains of Val173 and Ile251. Disruption of this interaction network in the cancer mutant R249S causes local misfolding and loss of DNA binding [35, 36]. As anticipated, the loss of the phenol group in the Y163C mutant resulted in the formation of a cavity, and the crystal structure of the mutant revealed the incorporation of two additional structural water molecules next to the mutation site that are stabilized by an extended hydrogen bond network. The guanidinium group of Arg249 was rotated by about 90° relative to its orientation in the wild type, thereby forming a stacking interaction with the imidazole ring of His168 while retaining the salt bridge with Glu171. This was accompanied by a flip of the Asn247 side chain, which formed a hydrogen bond to one of the two mutant-specific structural water molecules and the guanidium group of Arg249 (water molecule W1 in Fig. 4B). There were also notable structural shifts in the backbone of the L3 loop region from residues 242 to 249. Restraints on the backbone conformation in this region have been implicated in the impaired DNA-binding capacity of the cancer hotspot mutant G245S [17, 37].

We performed MD simulations to obtain insights into conformational fluctuations around the mutation-induced cavity. While the distance between the C_α_ atoms of the salt bridge forming residues Glu171 and Arg249 was fairly constant in the wild-type structure along the trajectory, there was a bimodal distribution in the Y163C mutant structure. Such a bimodal distribution in the mutant was also observed when monitoring selected distances along the newly formed cavity, indicating increased structural plasticity in the mutant. We specifically monitored the formation of the salt bridge and the interaction of Arg249 with His168, as this would drastically impact on the size and accessibility of the mutation-induced pocket. In both cases, the MD simulations showed a consistent broadening of the distance distribution in the mutant. From the time series, it is evident that the salt bridge can break and form in the mutant, while it remains mostly formed in the wild type, with much smaller distance fluctuations (Fig. 4D and Supplementary Fig. S2). A systematic analysis of structural snapshots with a broken salt bridge revealed two main populations: one where the Met246 side chain reaches into the mutation-induced pocket and one without rearrangement of Met246 where the pocket increased substantially in size (Fig. 4C, Table 3, and Supplementary Figs. S2C,S3B). This structural fluctuation is likely to modulate both the accessibility of the mutation-induced pocket for small molecules as well as the positioning of the DNA-contact residue Arg248 and surface complementarity for productive DNA binding.

### Response to ATO treatment correlates with the location of the mutation in the structure

**Fig. 5:**
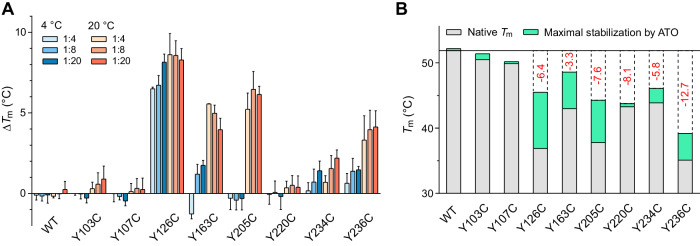
Effects of ATO treatment on mutant p53 DBD thermostability as measured by DSF. All measurements were performed in the stabilized quadruple mutant scaffold of the DBD. **A** The increase in the melting temperature for each mutant upon ATO treatment is given as Δ*T*_m_ = *T*_m_ (mutant after ATO treatment) - *T*_m_ (mutant control without ATO). The mutants were incubated for 16 h at either 4 or 20 °C, with protein:ATO ratios of 1:4, 1:8, and 1:20. The mean of at least three independent measurements with SD is given. **B** Maximal *T*_m_ value of the Tyr-to-Cys cancer mutants after incubation with ATO and relation to the mutation-induced stability loss in each case.

Many structural mutants were reported to be reactivated by ATO, which binds to a cryptic binding site in the loop-sheet-helix motif [12]. Here, we systematically quantified the effect on the thermostability of all Tyr-to-Cys DBD mutants by incubating them with ATO for 16 h at varying ratios and different temperatures, followed by DSF measurements (Fig. 5 and Supplementary Table S3). The effect on the melting temperature of the mutants varied significantly, ranging from essentially no ATO-induced stabilization (Y220C and the pseudo-wild type, QM) to an increase in *T*_m_ by up to 9 °C (Y126C and Y205C). For all mutants, however, the maximal stability gain by ATO treatment remained below the overall stability loss upon mutation. The cysteines targeted by ATO (Cys124, Cys135, and Cys141), even though located close to the surface, are either partly or totally buried in the available crystal structures of the p53 DBD. For ATO to bind, the loop-sheet-helix motif, therefore, has to exhibit a certain degree of structural plasticity. Accordingly, we consistently observed a much lower stabilization at 4 °C than at 20 °C, and essentially no stabilization for the non-mutated template. For the various Tyr-to-Cys mutants, there was an interesting proximity effect, and the data also suggest a conformational coupling across the DNA-binding surface and the zinc-binding loops. The strongest stabilization overall was observed for the Y126C mutant (particularly prominent in the series at low temperature) where the mutation site is directly next to the ATO binding site, which becomes more dynamic upon mutation and is, therefore, poised for interaction with ATO. An apparent proximity effect was also seen for the two internal cavity-creating mutations, Y234C and Y236C. Here, ATO had a much more stabilizing effect on Y236C (maximal stabilization by 4.1 ± 1.0 °C) than on Y234C (maximal stabilization by 2.2 ± 0.5 °C). The Y236C mutation is located closer to the ATO site than Y234C, but it also has to be taken into consideration that the Y236C mutant is much more severely destabilized overall by the point mutation. The Y163C and Y205C mutants in the L2/L3 loop region harboring the zinc-binding site were also significantly stabilized, with maximum Δ*T*_m_ values of 5.6 and 9.4 °C, respectively. Intriguingly, the thermolabile Y220C mutant showed virtually no stabilization by ATO treatment, indicating only minimal, if any, conformational coupling between the mutation site at the far end of the β-sandwich and the ATO binding site.

Our systematic stability measurements and structural studies on the Tyr-to-Cys cancer mutants provide a rationale for the differential phenotypic response upon ATO treatment reported for some of those mutants by Chen and colleagues [12]. In those experiments, the Y234C mutant showed both conformational and functional rescue, monitored with the conformation-specific antibody PAb1620 and by reporter gene assay, respectively. Y163C showed a conformational but no functional rescue, which can be explained by a substantial stabilization upon ATO treatment but local distortion of the L3 loop in the DNA-binding surface in the folded state that prevents sequence-specific DNA binding. Interestingly, the Y205C mutant was not conformationally rescued in cells, despite a substantial stabilization in our thermal-shift assay, indicating that a certain stability threshold must to be reached, which is more challenging for highly destabilized mutants such as Y205C than for Y163C or Y234C. It has, however, also to be noted that the binding epitope of PAb1620 in wild-type p53 includes Leu206 in the immediate environment of the Y205C mutation site [38]. A more recent study on ATO rescue from the same group classified Y126C as partially rescuable and Y236C as not rescuable [39], in perfect agreement with our stability data showing that the stabilization of the Y236C mutant by ATO is small compared with the overall stability loss upon mutation (Fig. 5B).

We also observed differential stabilization of the Tyr-to-Cys mutants upon modification with antimony compounds that noncovalently bind to the cryptic ATO binding site. The stabilization upon treatment with sodium stibogluconate was generally less pronounced than upon covalent modification with ATO, with maximal *T*_m_ shifts of about 5–6 °C (Supplementary Table S4), which would appear to be a relatively modest stability increase for functional rescue in cells, considering the large overall stability loss for some of those mutants. Consistent with our stability data, none of the Tyr-to-Cys mutants were identified as potentially treatable p53 mutations by potassium antimony tartrate in the cell-based assays by Tang et al. [13].

### Implications for the development of mutant p53 reactivators: mutant-specific vs generic stabilizers

Overall, our data provide intriguing insights into the structural, dynamic, and energetic effects of Tyr-to-Cys mutations in the p53 DBD, with important implications for mutant rescue strategies and the detection of ts mutants. Y234C and Y236C, which are destabilized via the formation of internal cavities, are classical ts mutants with the potential to be reactivated with generic stabilizers. However, due to the much higher stability loss in the Y236C mutant, reactivation may be more challenging for this particular mutant, requiring a much higher gain in stability through the action of chemical chaperones than for the Y234C mutant or other mutants for which a ts phenotype and partial reactivation have been shown in cells [10]. Also, the permissive temperature at which activity can be detected is expected to be drastically lower for the Y236C and similarly destabilized mutants than for the Y234C or Y220C mutant. The classical ts assay format in human cell lines comparing activities at 37 and 32 °C may, therefore, significantly underestimate the number of ts mutants and, hence also, the number of mutants that are potentially rescuable with chemical chaperones.

An interesting question in that context is how much a particular mutant has to be stabilized to elicit a therapeutic response. Our data on the Y220C mutant suggest that only partial recovery of the mutation-induced stability loss to a certain threshold stability is needed to restore p53 signaling pathways in cells [22, 23], which is further supported by a recent study on a DNA-binding impaired mutant showing that partial reactivation of p53 is sufficient for inducing cancer regression [40].

The differential stabilization achieved with ATO and the proximity effect observed for the different mutants provide a strong rationale for developing mutant-specific stabilizers alongside generic binders. Each of the Y126C, Y163C, and Y205C cancer mutations created characteristic alterations on the protein surface. However, the mutation-induced cavities for Y126C and Y205C are relatively small compared with the extended surface crevice in the already druggable Y220C mutant (Table 3). A special case is the Y163C mutant, which may also apply to other structural mutants, including the hotspot mutants G245S and R249S. Lack of temperature sensitivity [10] and local distortions in the DNA-binding interface of the Y163C mutant are a strong indication that merely stabilizing the protein may not be sufficient for functional rescue, unless there is a simultaneous, specific restoration of the local conformation of the DNA-binding surface around the mutation site. It is conceivable that this may also be achieved with allosteric binders. Although the mutation-induced pocket in the crystal structure of the Y163C mutant appears not that attractive a target at first sight due to its relatively small size, our MD simulations revealed a pronounced structural plasticity, with transient protein states that are characterized by a drastically enlarged binding pocket. Selected snapshots or ensembles from MD simulations [14, 41] may be exploited for virtual screening of small-molecule binders directly targeting the Y163C-induced cavity. Druggability prediction for the mutation-induced pocket in the MD snapshot shown in Fig. 4C using the program PockDrug [42] gave a reasonably high druggability score of 0.68 compared with a value of 0.90 for the druggable Y220C pocket. In addition, the Tyr-to-Cys mutation not only creates a cavity but also provides a handle for covalent modification with thiol-reactive compounds, thereby providing additional targeting opportunities, as shown recently for the Y220C mutant [43]. Future fragment screening campaigns on Y163C and other cavity-creating mutants are likely to further expand the druggable p53 mutome and inspire translational work to develop personalized anticancer drugs.

## Materials and methods

### Protein expression and purification

Mutant p53 DBDs were generated, expressed, and purified using standard purification techniques as described previously [25]. Briefly, the pET24a-based vectors used for recombinant expression in *E. coli* C41 encoded for a fusion protein containing an N-terminal hexahistidine tag, followed by the lipoyl domain of *Bacillus stearothermophilus* dihydrolipoamide acetyltransferase (Uniprot entry P11961, residues 2–85) for improved solubility, a TEV protease cleavage site, and the human p53 DBD variant of interest (residues 94–312). The DBD mutants were purified at 4 °C by Ni-NTA chromatography, followed by TEV protease cleavage overnight, affinity chromatography using a heparin column, and a final size-exclusion chromatography step on Superdex75.

### Protein crystallography and structure determination

Crystals of the Tyr-to-Cys mutants of the human p53 DBD (with stabilizing mutations M133L, V203A, N239Y, and N268D) were grown by vapor diffusion using the sitting drop technique. Detailed crystallization conditions and cryoprotectants used for each mutant are listed in Supplementary Table S5. Crystals were flash-frozen in liquid nitrogen, and X-ray datasets were collected at 100 K at beamline X06SA of the Swiss Light Source, Villigen, Switzerland. Diffraction data were integrated with the program XDS [44] and scaled with AIMLESS [45], which is part of the CCP4 package [46]. The structures were then solved by molecular replacement using PHASER [47] with PDB entry 1UOL or 6SHZ as a search model. The structures were refined using iterative cycles of manual model building in COOT [48] and refinement in PHENIX [49]. Dictionary files for buffer components were generated using the Grade Web Server (http://grade.globalphasing.org). The geometry of the final models was validated using MolProbity [50]. Data collection and refinement statistics are listed in Table 2. Structural figures were prepared using PyMOL (www.pymol.org).

### Differential scanning fluorimetry (DSF) measurements

Melting temperatures, *T*_m_ values, of p53 mutant DBDs were determined by DSF using an Agilent MX3005P real-time qPCR instrument (excitation/emission filters = 492/610 nm). Measurements were performed in a 96-well plate with an assay buffer consisting of 25 mM HEPES, pH 7.5, 500 mM NaCl, and 0.5 M TCEP, with a final protein concentration of 5 μM. The fluorescent dye SYPRO Orange (5000×, Invitrogen) was added at a dilution of 1:1000 (total volume of 20 μL per well). The fluorescence signal was then monitored upon temperature increase from 25 to 80 °C, at a heating rate of 3 °C/min, and *T*_m_ values were calculated after fitting the fluorescence curves to the Boltzmann function. For measuring the effects of ATO and stibogluconate treatment on protein stability, the DBDs were incubated at 4 and 20 °C in assay buffer with different concentrations of compound prior to the addition of SYPRO Orange; the incubation time was 16 h for ATO and 2 or 16 h for stibogluconate. All DSF measurements were performed in at least three independent experiments, and *T*_m_ values are given as the mean with standard deviation.

### Molecular dynamics simulations

Molecular dynamics (MD) simulations were set up for the DBD of stabilized pseudo-wild-type p53 (M133L/V203A/N239Y/N268D) and the corresponding structures of the Y126C and Y163C mutant. As starting models, PDB entry 1UOL and the mutant crystal structures determined in this study were used (chain A in each case). Disordered loops in the crystal structure of the Y126C mutant were modeled before the MD simulation. Bound buffer molecules, glycerol or ethylene glycol from the crystallization/cryo conditions were removed from the calculations. The starting models were solvated in truncated octahedron simulation boxes with walls at least 1.2 nm away from the protein. The TIP3P [51] model was used for the explicit simulation of water molecules. The system was neutralized and taken to physiological ion concentration (0.15 M) by changing some water molecules into sodium and chlorine ions. Simulations were carried out with the CHARMM36m force field [52] and the program GROMACS.2018 [53]. The zinc ion and the coordinating cysteine/histidine residues were modeled according to Budiman et al. (2007) and Godwin et al. (2017) [54, 55]. The resulting systems comprised between 35787 and 38845 atoms. The temperature of the system was kept constant at 300 K using a Nosé-Hoover thermostat [56, 57] with τ_t_ = 1 ps, and the pressure was kept constant at 1 bar with the Parrinello-Rahman algorithm [58] with τ_p_ = 1 ps. Direct non-bonded interactions were cut off at 1.2 nm with a switch function on Van der Waals interactions starting at 1.0 nm. Long-range electrostatic interactions were computed using sPME [59]. Covalent bonds with hydrogen atoms were constrained with the LINCS algorithm [60], and the integration time step was set to 2 fs. The systems were minimized for max 50,000 steps and equilibrated in the NVT ensemble for 1 ns with positional restraints on the positions of the heavy atoms of the protein. Then, they were further equilibrated for 1 ns in the NPT ensemble without restraints. For each DBD variant, four 200-ns production runs were performed. The MD trajectories were analyzed using VMD [61] and WORDOM [62].

### Supplementary information

Supporting Information

## Data Availability

The atomic coordinates and structure factors of the p53 DNA-binding domain mutants have been deposited in the Protein Data Bank (PDB) under accession codes 8QWK (Y126C), 8QWL (Y163C), 8QWM (Y205C), 8QWN (Y220C), 8QWO (Y234C), and 8QWP (Y236C).
