# Supplementary material for: Structural basis of p53 inactivation by cavity-creating cancer mutations and its implications for the development of mutant p53 reactivators
Source: Cell Death Dis. 2024 Jun 11;15(6):408. doi: 10.1038/s41419-024-06739-x (PMC11166945; doi:10.1038/s41419-024-06739-x)
Supplement: Supplementary file 1 — Supporting Information [file 41419_2024_6739_MOESM1_ESM.pdf]

Supporting Information

Structural basis of p53 inactivation by cavity-creating cancer mutations and its implications for the development of mutant p53 reactivators

Dimitrios-Ilias Balourdas[1,2], Anja M. Markl[1], Andreas Krämer[1,2], Giovanni Settanni[3,4], Andreas C. Joerger[1,2]*

[1]Institute of Pharmaceutical Chemistry, Goethe University, Max-von-Laue-Str. 9, 60438 Frankfurt am Main, Germany

[2]Structural Genomics Consortium (SGC), Buchmann Institute for Life Sciences, Max-von-Laue-Str. 15, 60438 Frankfurt am Main, Germany

[3]Faculty of Physics and Astronomy, Ruhr University Bochum, Universitätsstr. 150, 44801 Bochum, Germany

[4]Physics Department, University of Mainz, Staudingerweg 7, 55099 Mainz, Germany

Table S1. Frequency of DBD Tyr mutations in the IARC/NCI TP53 Database Release R20 (July 2019)[1]

Residue	Number of cases[2] of mutation to					
	Cys	His	Asn	Ser	Asp	Phe
Tyr103	0	0	0	0	0	0
Tyr107	1	1	0	0	4	0
Tyr126	22	3	7	3	7	1
Tyr163	166	29	25	5	5	1
Tyr205	122	12	4	20	19	10
Tyr220	402	20	18	17	4	1
Tyr234	147	31	16	8	6	1
Tyr236	88	15	20	3	10	2

[1] N = 28,866; https://tp53.isb-cgc.org

[2] In addition, there is one case of a Y126G, Y234K, and Y234Q mutation.

Table S2. Frequency of DBD Tyr mutations in the UMD TP53 Mutation Database[1]

Residue	Number of cases[2] of mutation to					
	Cys	His	Asn	Ser	Asp	Phe
Tyr103	0	1	0	0	0	0
Tyr107	3	6	0	1	20	0
Tyr126	98	22	29	9	38	3
Tyr163	508	78	58	12	15	2
Tyr205	293	51	18	41	49	24
Tyr220	1260	68	53	73	20	3
Tyr234	405	116	48	23	32	2
Tyr236	267	45	49	18	35	2

[1] Release 2017_R2 (N = 80,402); https://p53.fr/tp53-database

[2] In addition, there is one case of a Y126G and Y234R mutation

Table S3. Thermal stabilization of p53 mutant DBDs with arsenic trioxide

Tyr mutation	ΔT_m (°C)[1]					
	4 °C			20 °C		
	1:4 ratio	1:8 ratio	1:20 ratio	1:4 ratio	1:8 ratio	1:20 ratio
WT (QM)	-0.1 ± 0.3	-0.2 ± 0.3	-0.1 ± 0.5	-0.2 ± 0.1	0.0 ± 0.3	0.3 ± 0.5
WT (TM)	-0.1 ± 0.3	0.2 ± 0.3	0.4 ± 0.5	0.2 ± 0.4	0.8 ± 0.7	1.2 ± 1.0
Y103C	0.0 ± 0.1	0.0 ± 0.3	-0.3 ± 0.3	0.3 ± 0.4	0.6 ± 0.7	0.9 ± 0.8
Y107C	0.0 ± 0.5	-0.2 ± 0.2	-0.5 ± 0.3	0.1 ± 0.5	0.3 ± 0.6	0.3 ± 0.7
Y126C	6.5 ± 0.1	6.7 ± 0.6	8.2 ± 0.5	8.6 ± 1.3	8.6 ± 0.9	8.3 ± 0.7
Y163C	-1.3 ± 0.3	1.2 ± 0.6	1.7 ± 0.3	5.6 ± 0.1	5.0 ± 0.5	4.0 ± 0.7
Y205C (QM)	-0.3 ± 0.7	-0.4 ± 0.6	-0.3 ± 0.7	5.2 ± 1.0	6.5 ± 1.1	6.1 ± 0.5
Y205C (TM)	0.0 ± 0.2	0.7 ± 1.4	2.8 ± 0.7	9.4 ± 0.3	9.4 ± 0.2	9.6 ± 0.9
Y220C	-0.1 ± 0.6	0.1 ± 0.7	-0.2 ± 0.8	0.4 ± 0.4	0.5 ± 0.6	0.4 ± 0.7
Y234C	0.2 ± 0.5	0.7 ± 0.8	1.4 ± 0.6	0.7 ± 0.4	1.6 ± 0.8	2.2 ± 0.5
Y236C	0.6 ± 0.6	1.4 ± 0.8	1.5 ± 0.2	3.3 ± 1.5	4.0 ± 1.2	4.1 ± 1.0

[1]ΔT_m= T_m (protein after ATO treatment) - T_m (protein without treatment). Buffer: 25 mM HEPES, pH 7.5, 500 mM NaCl. Mean ± SD of four independent measurements is given (each performed in technical triplicates). Protein concentration was 5 µM and arsenic trioxide concentrations were 20 µM, 40 µM, and 100 µM, respectively (1:4, 1:8, and 1:20 ratio). Samples were incubated at either 4 or 20 °C for 16 h, prior to fluorescence measurement.

Table S4. Thermal stabilization of p53 mutant DBDs with sodium stibogluconate

	ΔT_m (°C)[1]			
Tyr mutation	2 h<br>1:20 ratio	2 h<br>1:200 ratio	16 h<br>1:20 ratio	16 h<br>1:200 ratio
WT (QM)	0.4 ± 0.3	0.3 ± 0.2	0.2± 0.1	0.3 ± 0.1
Y126C	1.2 ± 0.2	2.3 ± 0.3	0.4 ± 0.2	2.0 ± 0.4
Y163C	1.4 ± 0.2	2.3 ± 0.3	2.5 ± 0.6	6.2 ± 0.5
Y205C	1.5 ± 0.3	3.3 ± 0.5	2.2 ± 0.3	5.7 ± 0.2
Y220C	0.2 ± 0.3	-0.1 ± 0.1	-0.1 ± 0.1	-0.3 ± 0.1
Y234C	0.3 ± 0.8	0.5 ± 0.3	0.8 ± 0.3	2.0 ± 0.2
Y236C	1.1 ± 0.5	2.1 ± 0.2	0.9 ± 0.4	3.2 ± 0.1

[1]ΔT_m= T_m (protein after sodium stibogluconate treatment) - T_m (protein without treatment). Buffer: 25 mM HEPES, pH 7.5, 500 mM NaCl. Mean ± SD of three independent measurements is given (each performed in technical triplicates). Protein concentration was 5 µM and sodium stibogluconate concentrations were 100 µM and 1 mM, respectively (1:20 and 1:200 ratio). Samples were incubated at 20 °C for 2 h or 16 h, prior to fluorescence measurement.

Table S5. Crystallization conditions of DBD mutants

Mutant	Temperature	Reservoir buffer	Protein buffer
Y126C	20 °C	0.7 M sodium citrate, 0.1 M bis-tris-propane (pH 7.0)	25 mM HEPES (pH 7.5), 300 mM NaCl, 0.5 mM TCEP
Y163C	4 °C	24% PEG 3350 (w/v), 10% ethylene glycol (v/v), 0.2 M sodium malonate (pH 7.0)	25 mM HEPES (pH 7.5), 200 mM NaCl, 0.5 mM TCEP
Y205C-TM	4 °C	18% PEG 3350 (w/v), 15% ethylene glycol (v/v), 0.2 M Na/K tartrate	25 mM HEPES (pH 7.5), 200 mM NaCl, 0.5 mM TCEP
Y220C	20 °C	30% w/v PEG 4000 (w/v), 0.1M Tris pH 8.5, 0.2 M magnesium chloride hexahydrate	25 mM HEPES (pH 7.5), 200 mM NaCl, 0.5 mM TCEP
Y234C	20 °C	19% PEG 4000 (w/v), 0.1 M HEPES (pH 7.0), 5 mM DTT	25 mM phosphate (pH 7.5), 150 mM NaCl, 0.5 mM TCEP
Y236C	4 °C	19% PEG 3350 (w/v), 5% ethylene glycol (v/v), 0.2 M Na/K tartrate	25 mM HEPES (pH 7.5), 200 mM NaCl, 0.5 mM TCEP

For cryo protection of the crystals, the mother liquor was complemented with either 20 % (v/v) glycerol (Y234C) or 23% (v/v) ethylene glycol (all remaining mutants).

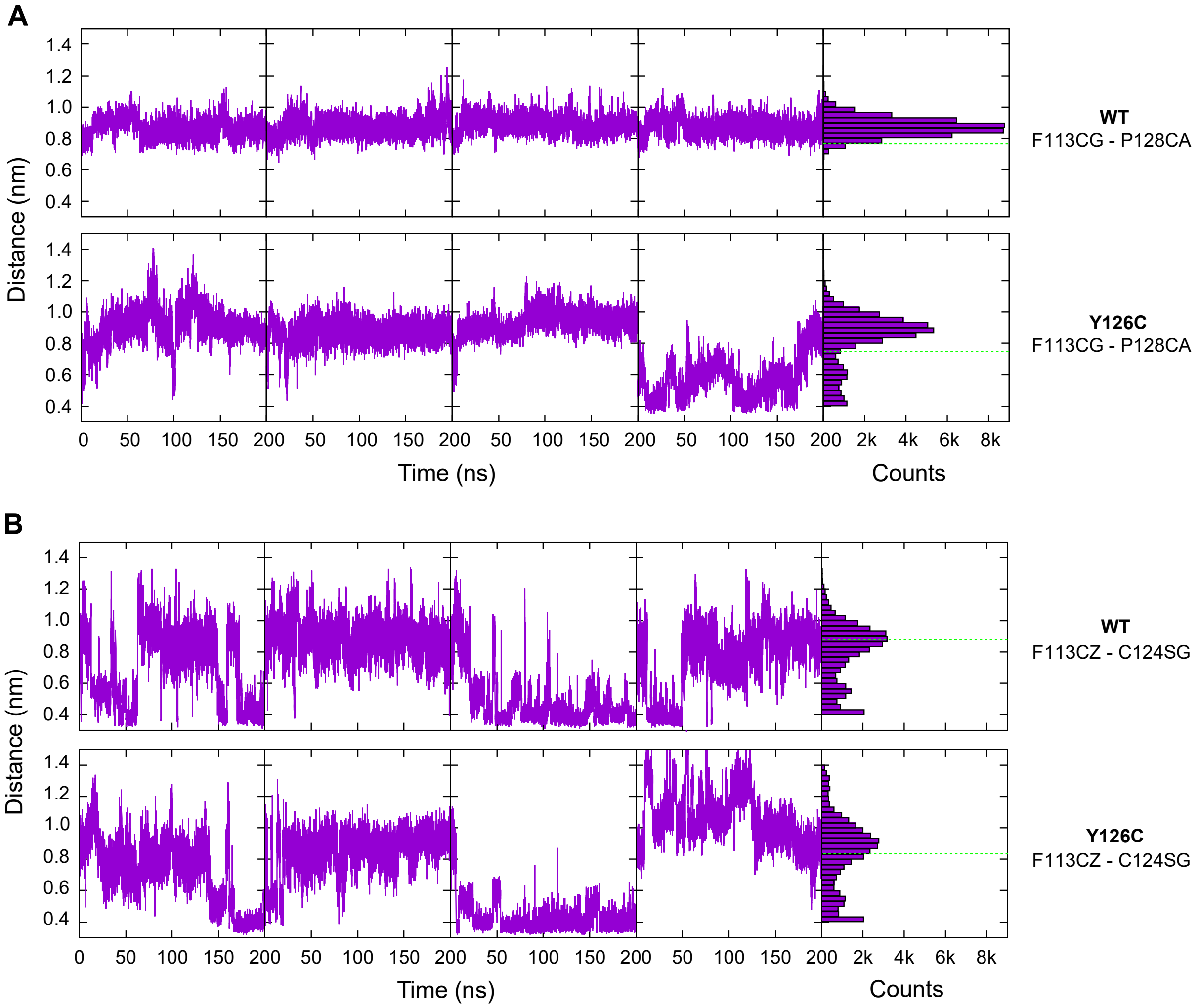

Figure S1. MD simulation of the p53 cancer mutant Y126C DBD. (A) Distance between the CG atom of Phe113 and the CA atom of Pro128 as a function of time over all MD simulations, showing a multimodal distribution in the Y126C mutant. (B) Distance between the CZ atom of Phe113 and the SG atom of Cys124 as a function of time over all MD simulations, revealing a flip of the Phe113 side chain in the third simulation of the WT and mutant structure (see also a representative structure in Figure S3A). The green line in each plot indicates the distance in the corresponding crystal structure ("WT" = chain A of PDB entry 1UOL).

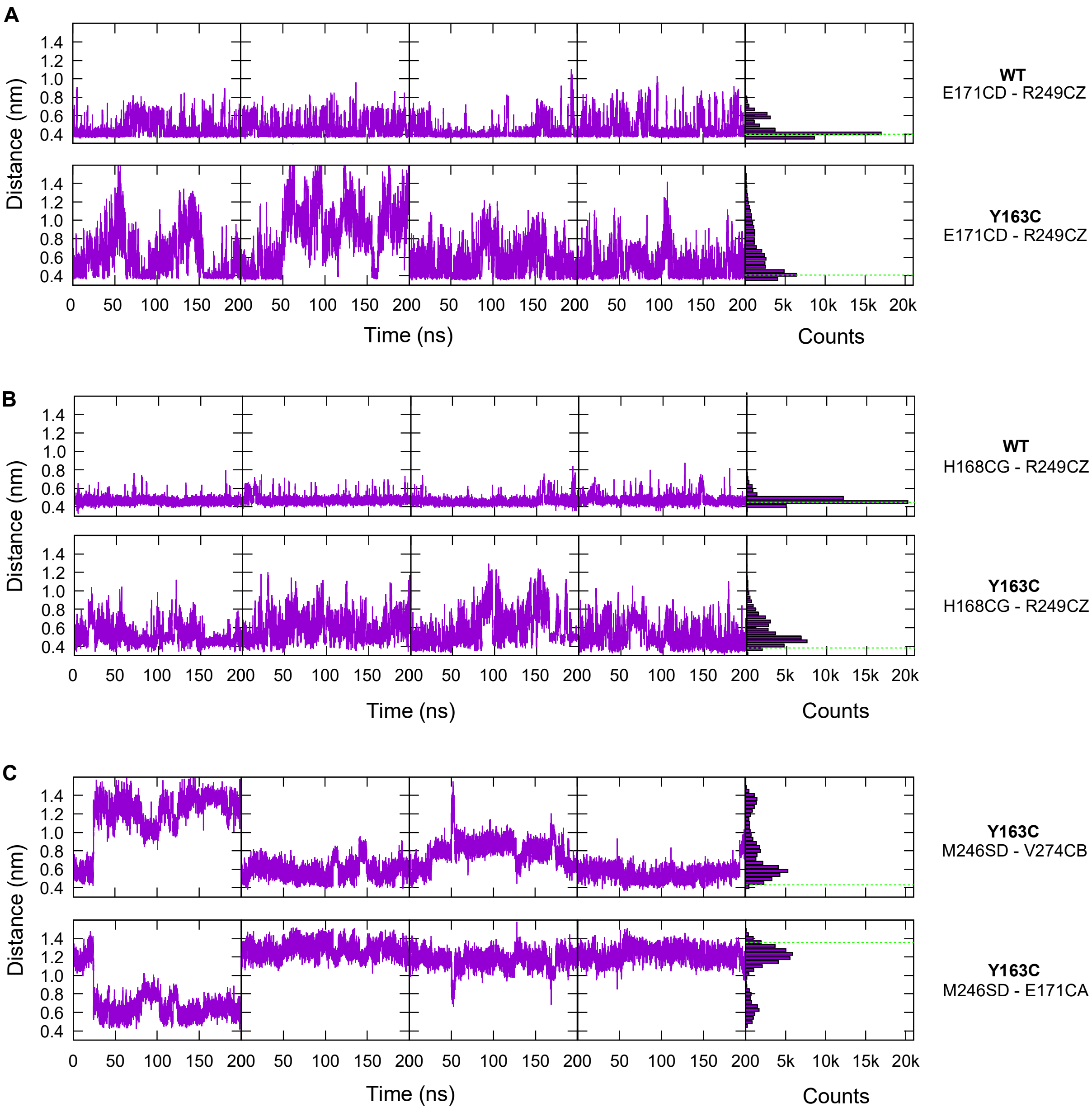

Figure S2. MD simulation of p53 cancer mutant Y163C DBD. (A) Distance between the CZ atom of Arg249 and the CD atom of Glu171 as a function of time over all MD simulations. The data show that the Glu171-Arg249 salt bridge can break and re-form in the Y163C mutant along the trajectory but is much more stable in the wild-type structure. (B) Distance between the CZ atom of Arg249 and the CG atom of His168 as a function of time over all MD simulations, showing a pronounced fluctuation in the Y163C mutant that is not seen for the WT. (C) Distances between Met246 and Glu171 or Val274 indicating a repacking of Met246 in the first simulation of the Y163C mutant (see also representative structures in Figure S3B). The green line in each plot indicates the distance in the corresponding crystal structure ("WT" = chain A of PDB entry 1UOL).

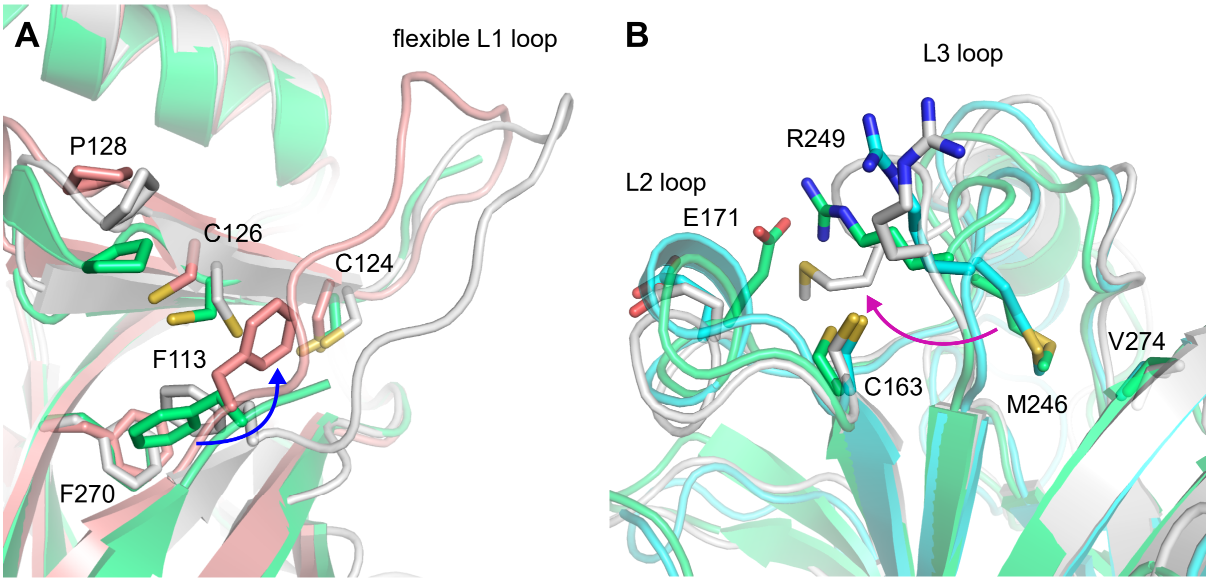

Figure S3. Structural fluctuations of p53 cancer mutants in MD simulations. (A) Crystal structure of the Y126C mutant (green) superimposed onto two different snapshots from the MD simulation of this structure (gray and pink structures): the MD snapshot in gray is similar to the crystal structure, whereas in the other snapshot (pink), the size of the mutation-induced pocket is increased through a flip of the aromatic ring of Phe113 toward Cys124 (cf. Phe113-Cys124 distance in Figure S1). (B) Plasticity of the Y163C-induced pocket in MD simulations. Superimposition of the Y163C crystal structure (green) and two representative snapshots from the Y163C mutant MD simulation with broken Glu171-Arg249 salt bridge seen along the trajectory (cyan and gray structures). The cyan structure corresponds to the opening up of the mutation-induced pocket shown in Figure 4C (snapshot f35486, time point at 106.88 ns of MD run no. 4), whereas the structure in gray is characterized by additional rearrangements in the L3 loop (snapshot f2707, time point at 53.94 ns of MD run no. 1). In the latter structure, the Met246 side chain has moved towards the backbone of Glu171, thereby constraining that part of the pocket but opening up the pocket elsewhere around the original Met246 binding site. Flipping of Met246 occurred independent of breaking/formation of the Glu171-Arg249 salt bridge in one of the MD simulations as shown by monitoring the distance between the Met246 sulfur atom and residues Val274 and Glu171, respectively, along the MD trajectory (Figure S2C). Interestingly, loss of the Glu171-Arg249 salt bridge in the R249S cancer mutant also affected the position of Met246. The crystal structure of this mutant (PDB entry 2BIO) revealed a misfolded conformation of the L3 loop where the usually solvent-exposed side chain of Met243 displaced Met246 from its hydrophobic environment next to the zinc-binding region.
